# Employee expectations towards employer-provided workplace health promotion and mental health support: a 2025 cross-sectional survey

**DOI:** 10.3389/fpubh.2026.1806781

**Published:** 2026-04-09

**Authors:** Agata Olearczyk, Kuba Sękowski, Mateusz Jankowski, Mariusz Gujski, Justyna Grudziąż-Sękowska

**Affiliations:** 1 Health Innovation Unit, SGH Warsaw School of Economics, Warsaw, Poland; 2 School of Public Health, Centre of Postgraduate Medical Education, Warsaw, Poland; 3 VIZJA University, Warsaw, Poland

**Keywords:** employees’ health, mental health, Poland, public health, workforce health, workplace health promotion

## Abstract

**Introduction:**

Workplace health promotion (WHP) interventions are public health activities targeted to working adults that may improve health outcomes, organizational performance, and work ability. This study aimed to assess employees’ expectations towards employer-provided workplace health promotion and mental health support, as well as to identify factors associated with employees’ expectations towards WHP and mental health support.

**Methods:**

This nationwide questionnaire-based cross-sectional study was conducted in Poland between 1 and 9 December 2025 among 1,030 full-time employees aged 18–60 years. Data were collected through computer-assisted web interviews (CAWI).

**Results:**

Out of all respondents (*n* = 1,030), 86.5% declared that employers should provide workplace health promotion activities. Most respondents (35% “definitely yes” and 36.1% “rather yes”) declared that employers should actively support employees’ mental health. Burnout prevention (35.7%), prevention of spine and musculoskeletal disorders (34.6%), and stress management strategies (34%) were the most frequently indicated WHP expected by employees. Having higher education (aOR: 2.13; 95%CI: 1.46–3.11; *p* < 0.001), living in cities from 20,000 to 99,999 residents (aOR: 2.18; 95%CI: 1.21–3.93; *p* = 0.01), being in informal relationship (aOR: 2.01; 95%CI: 1.08–3.75; *p* = 0.03), and working in companies with 250 employees or over (*p* = 0.02) were associated with employees’ expectations regarding employer-provided WHP activities. Number of employees was the only factor significantly associated with employees’ expectations regarding employer-provided mental health support (*p* < 0.05).

**Conclusion:**

Most full-time employees in Poland expect employers to implement workplace health promotion and to provide mental health support. Expectations varied by sociodemographic and job-related characteristics. These findings indicate high acceptability of WHP among employees and point to the need for scalable approaches that also reach workers in smaller companies.

## 1 Introduction

The World Health Organization (WHO) identifies the workplace as a key setting for implementing public health interventions (1). Workplaces provide access to large populations of working-age adults, as well as those difficult to reach through healthcare and preventive services (1, 2). Health-related behaviors can be shaped in the workplace through educational activities, supportive resources, organizational culture, and leadership practices (3–5).

The “Luxembourg Declaration on Workplace Health Promotion in the European Union” defines workplace health promotion (WHP) as combined efforts of employers, employees, and society to improve the health and well-being of people at work (3). WHP can be effectively achieved through a combination of improving the work organization and working environment, promoting active participation, and encouraging personal development (3).

Workplace health promotion is a voluntary employer activity that goes beyond legal obligations such as occupational health and safety measures, as well as occupational medicine (1, 3). In the European Union, employers are legally obligated to provide workplace safety measures, occupational risk mitigation, and initial, periodic and control occupational health examinations (4). WHP, on the other hand, encompasses a wide range of voluntary public health interventions, such as health promotion, prevention, well-being, mental health, ergonomics, physical activity, vaccination campaigns, and lifestyle-related behaviors (5–8). WHP is often positioned as an added employee benefit, reflecting an employer’s commitment to long-term workforce well-being (5, 9).

In recent years, mental health has gained priority in numerous WHP activities (1, 10, 11). Work-related stress, burnout, and mental health disorders significantly influence the productivity, absenteeism, presenteeism, and quality of life of employees (11, 12). WHP interventions on mental health support may include stress management programs, access to psychological consultations, psychotherapy, peer-support groups, and digital e-health interventions based on mobile applications and educational web-based platforms (10–13).

The results of systematic reviews and meta-analyses show that implementing workplace health promotion (WHP) can improve health outcomes, organizational performance, work ability, and reduce presenteeism and the number of sick leaves (3–10). However, the effectiveness of individual WHP interventions varies greatly (4–10). The effects of WHP interventions depend significantly on program design, implementation, group fit, communication, organizational culture, employee engagement, and participation (4–10, 14, 15). A review of the scientific literature does not allow for the identification of a single, universal model for WHP interventions. Planning and implementing WHP interventions requires adaptation to the specific characteristics of the employer (especially company size and type) as well as the needs and expectations of employees (14, 15).

Suboptimal uptake among employees is a key barrier to obtaining high effectiveness of workplace health promotion interventions (5, 6, 16). Participation in WHP is determined both by individual factors and organizational determinants (4–15). Lack of time, low motivation, perceived need, thematic matching, and health belief are key personal determinants influencing WHP uptake (5, 10, 14, 15, 17). Organizational climate, support from superiors, logistic barriers, communication quality, availability during working hours, equal access for manual and non-manual workers, confidentiality, co-payment policies, and fit with job demand are major organizational determinants of WHP interventions (14, 15, 17). Measuring employee expectations and preferences is a critical step towards designing effective WHP interventions (4–10). WHP targeting employees’ expectations, addressing health issues relevant to the target population, along with an organizational framework enabling equal and fair participation, determines the effectiveness of health-promoting activities in the workplace. Thus, monitoring of employees’ expectations towards WHP interventions is an important part of WHP planning and implementation (18).

There is a limited number of papers on workplace health promotion in Poland (19–24). Previous studies conducted in Poland have primarily focused on workplace health promotion from the employers’ or organizational perspective (19–22). Employers in Poland are obligated by law to ensure safe working conditions and access to occupational medicine. WHP in Poland is voluntary, and the scope of WHP interventions varies across company types and sectors (19, 20, 24). Due to the population aging and growing burden of chronic diseases, activities targeted on workforce health and well-being are particularly important for economic sustainability and development (25). However, there is limited evidence regarding employees’ expectations concerning workplace health promotion activities at the national level. Understanding employees’ expectations may provide valuable insights for the development of more effective and acceptable workplace health promotion strategies. Epidemiological data on employees’ expectations towards employer-provided workplace health promotion and mental health support may inform employers, policymakers, and public health specialists on future directions of workplace health promotion development in Poland. Based on previous literature on workplace health promotion and employee health expectations, the authors hypothesized that employees’ expectations regarding workplace health promotion activities are associated with sociodemographic characteristics, and job-related characteristics, which should be taken into consideration by employers while planning effective WHP.

Therefore, this study aimed to assess employees’ expectations towards employer-provided workplace health promotion and mental health support, as well as to identify factors associated with employees’ expectations towards workplace health promotion and mental health support.

## 2 Materials and methods

### 2.1 Study design and population

This nationwide questionnaire-based cross-sectional study was conducted in Poland between 1 and 9 December 2025 among 1,030 full-time employees aged 18–60 years.

Data were collected using a computer-assisted web interview (CAWI) approach. To obtain nationwide sample of full-time employees, a public opinion survey company (the Nationwide Research Panel Ariadna (26)) was contracted for data collection on behalf of authors who provided all scientific background and procedures guidance.

Participants were recruited from a pool of registered and verified The Nationwide Research Panel Ariadna members (over 100,000 individuals) (26). A quota-based sampling strategy was applied. Stratification model included gender (2 categories), age (5 categories) and place of residence (5 categories). The stratification model included 50 quota strata. Quota controls were jointly set to approximate the structure of the Polish adult population by gender, age, and place of residence, following the Demographic Yearbook of the Republic of Poland, published annually by the Statistics Poland (27).

After the literature review on workplace health promotion activities, with particular emphasis on Poland, the inclusion criteria for the study population included: age 18–60 years, professionally active, and full-time employment under an employment contract. Individuals aged over 60 years, part-time employers, self-employed, or those employed under the civil contract were excluded from the study. Retirement age in Poland is 60 years for women and 65 for men. In order to ensure comparable data between genders, the exclusion criterion for all genders was age over 60 years. A full-time employment contract is the most frequently chosen form of employment in Poland.

Respondents selected from the Nationwide Research Panel Ariadna members based on sampling criteria received personalized invitation to participate in this study and URL link to the study questionnaire via text message and e-mail. If selected respondent refused to participate, the next one was selected based on the stratification model and quota strata. Respondents were obligated to fill out all the questions, so there were no missing respondents. The Nationwide Research Panel Ariadna response rate is estimated at 22%.

The similar research methods were used in other nationwide studies from Poland (28–30).

### 2.2 Study instrument

The questionnaire was developed by the research team based on review of the scientific literature on workplace health promotion and mental health support in workplace as well as previously implemented WHP programs (1, 3, 5–10, 14). The selection of questionnaire domains focused on health promotion areas commonly addressed in workplace settings, including mental health support, lifestyle-related behaviors (e.g., physical activity and healthy nutrition), and prevention of work-related health risks. Based on this review, a list of 16 potential workplace health promotion domains was developed and subsequently included in the questionnaire.

Each respondent received the following information, before filling out the questionnaire: Workplace health promotion (WHP) is a coordinated set of activities and strategies at the workplace (offered by the employer) to encourage the health and safety of all employees. WHP is not occupational medicine, but additional activities such as promoting a healthy lifestyle, especially a healthy diet, physical activity, and disease prevention.

Employee expectations towards employer-provided workplace health promotion and mental health support were assessed using the following questions: (1) In your opinion, should the employer implement workplace health promotion (WHP) activities? (yes/no); (2) In your opinion, should employers actively support employees’ mental health (e.g., through consultations with a psychologist or psychiatrist, access to psychotherapy, mobile apps, educational materials, etc.)? (5-point Likert scale); and (3) Which areas of workplace health promotion would you like to participate in? Please select all that apply or specify other (16 different health-related topics). The full questionnaire is provided in Supplementary File S1.

Prior to the main study, a pilot test was conducted among 11 working-age adults (18–60 years), who completed the questionnaire twice with a 7–10-day interval. Following this procedure, two items were revised and four response options were refined to improve readability and consistency. Kappa coefficients for the critical questions ranged from 0.78 to 0.96.

### 2.3 Ethics statement

The study protocol was reviewed and approved by the Ethics Committee at the Centre for Medical Postgraduate Education (approval no. 99/2025, dated 19 November 2025). All the procedures followed the Declaration of Helsinki principles. Participation in this study was voluntary, anonymous, and all participants declared informed consent.

### 2.4 Statistical analysis

All analyses were performed using IBM SPSS Statistics v.29 (IBM, Armonk, NY, USA). Categorical variables were reported as frequencies and percentages, and differences between groups were assessed using chi-square tests based on cross-tabulations.

Multivariable logistic regression analyses were performed to identify: (model 1) factors associated with employees’ expectations regarding employer-provided workplace health promotion (WHP) activities; and (model 2) factors determining the belief that employers actively support employees’ mental health. Each explanatory variable was first evaluated in bivariable models, and variables reaching statistical significance were subsequently entered into a multivariable logistic regression model. The strength of association was measured by odds ratio (OR) with 95% confidence interval (95% CI). Statistical significance was set at *p* < 0.05. In multivariable logistic regression adjusted odds ratio (aOR) was presented.

## 3 Results

Detailed characteristics of the study population, including socio-demographic characteristics and characteristics of the respondents’ workplace, are presented in Table 1. Overall, 86.5% of respondents declared that employers should provide workplace health promotion (WHP) activities (Table 1). Respondents with higher education (90.5%) more often declared that employers should provide WHP activities compared to those without higher education (*p* < 0.001). Residents of rural areas less frequently indicated that employers should provide WHP activities compared to residents of urban areas (*p* = 0.03). Respondents who were in informal relationships more frequently declared that employers should provide WHP activities compared to groups with other marital statuses (p = 0.03). The percentage of respondents declaring that the employer should provide WHP activities increased with the company’s headcount (*p* = 0.02) (Table 1).

**Table 1:** Characteristics of the study population by employees’ expectations regarding employer-provided workplace health promotion (WHP) activities (*n* = 1,030).

Variable	Overall		In your opinion, should the employer implement workplace health promotion (WHP) activities?				
			Yes		No		
	*n*	%	*n*	%	*n*	%	*p*
Gender							
Female	521	50.6	457	87.7	64	12.3	0.3
Male	509	49.4	434	85.3	75	14.7	
Age [years]							
18–24	52	5.0	47	90.4	5	9.6	0.5
25–34	317	30.8	279	88.0	38	12.0	
35–44	284	27.6	248	87.3	36	12.7	
45–54	266	25.8	222	83.5	44	16.5	
55–60	111	10.8	95	85.6	16	14.4	
Educational level							
Higher	588	57.1	532	90.5	56	9.5	**<0.001**
Less than higher	442	42.9	359	81.2	83	18.8	
Place of residence							
Rural area	351	34.1	290	82.6	61	17.4	**0.03**
City below 20,000 residents	136	13.2	116	85.3	20	14.7	
City from 20,000 to 99,999 residents	198	19.2	182	91.9	16	8.1	
City from 100,000 to 499,999 residents	201	19.5	178	88.6	23	11.4	
City ≥ 500,000 residents	144	14.0	125	86.8	19	13.2	
Marital status							
single	274	26.6	234	85.4	40	14.6	**0.03**
Married	529	51.4	451	85.3	78	14.7	
Informal relationship	207	20.1	191	92.3	16	7.7	
Other (divorced or widowed)	20	1.9	15	75.0	5	25.0	
Having children <18 y.o.							
Yes	420	40.8	368	87.6	52	12.4	0.4
No	610	59.2	523	85.7	87	14.3	
Number of household members							
1 (living alone)	152	14.8	134	88.2	18	11.8	0.7
2	286	27.8	244	85.3	42	14.7	
3 or more	592	57.4	513	86.7	79	13.3	
Workplace							
Type of company							
Private Polish company	513	49.8	432	84.2	81	15.8	0.3
Public institution	250	24.3	222	88.8	28	11.2	
Private foreign-owned company	205	19.9	183	89.3	22	10.7	
State-owned enterprise	48	4.7	41	85.4	7	14.6	
Non-governmental organization	14	1.4	13	92.9	1	7.1	
Number of employees							
Up to 9	119	11.6	93	78.2	26	21.8	**0.02**
10–49	240	23.3	204	85.0	36	15.0	
50–249	289	28.1	250	86.5	39	13.5	
250–500	108	10.5	98	90.7	10	9.3	
More than 500	274	26.6	246	89.8	28	10.2	
Job satisfaction							
Not at all satisfied	61	5.9	53	86.9	8	13.1	0.5
Rather dissatisfied	176	17.1	157	89.2	19	10.8	
Rather satisfied	630	61.2	545	86.5	85	13.5	
Very satisfied	163	15.8	136	83.4	27	16.6	
Main type of work							
Manual work	369	35.8	310	84.0	59	16.0	0.08
Non-manual work	661	64.2	581	87.9	80	12.1	
Job position							
Entry-level	398	38.6	338	84.9	60	15.1	0.7
Specialist	462	44.9	403	87.2	59	12.8	
Manager	123	11.9	109	88.6	14	11.4	
Director-level	24	2.3	22	91.7	2	8.3	
Executive	23	2.2	19	82.6	4	17.4	
Working time arrangement							
Fixed daytime hours	603	58.5	521	86.4	82	13.6	0.5
Shift work without night shifts	118	11.5	102	86.4	16	13.6	
Shift work including night shifts	99	9.6	81	81.8	18	18.2	
Flexible working hours	138	13.4	124	89.9	14	10.1	
Task-based working time	72	7.0	63	87.5	9	12.5	

Statistically significant values are bolded.

### 3.1 Factors associated with employees’ expectations regarding employer-provided workplace health promotion (WHP) activities

In multivariable logistic regression model (Table 2), having higher education (aOR: 2.13; 95%CI: 1.46–3.11; *p* < 0.001), living in cities from 20,000 to 99,999 residents (aOR: 2.18; 95%CI: 1.21–3.93; *p* = 0.01), being in informal relationship (aOR: 2.01; 95%CI: 1.08–3.75; *p* = 0.03), and working in companies with 250 employees or over (*p* = 0.02) were associated with employees’ expectations regarding employer-provided workplace health promotion activities (Table 2).

**Table 2:** Factors associated with employees’ expectations regarding employer-provided workplace health promotion activities (*n* = 1,030).

In your opinion, should the employer implement workplace health promotion (WHP) activities?—yes				
Variable	Bivariable logistic regression		Multivariable logistic regression	
	OR (95% CI)	*p*	aOR (95% CI)	*p*
Gender				
Female	1.23 (0.86–1.77)	0.3		
Male	Reference			
Age [years]				
18–24	1.58 (0.55–4.59)	0.4		
25–34	1.24 (0.66–2.32)	0.5		
35–44	1.16 (0.62–2.19)	0.6		
45–54	0.85 (0.46–1.58)	0.6		
55–60	Reference			
Educational level				
Higher	2.20 (1.53–3.16)	**<0.001**	2.13 (1.46–3.11)	**<0.001**
Less than higher	Reference		Reference	
Place of residence				
Rural area	Reference		Reference	
City below 20,000 residents	1.22 (0.71–2.11)	0.5	1.14 (0.65–2.00)	0.6
City from 20,000 to 99,999 residents	2.39 (1.34–4.28)	**0.003**	2.18 (1.21–3.93)	**0.01**
City from 100,000 to 499,999 residents	1.63 (0.97–2.72)	0.06	1.29 (0.76–2.20)	0.3
city ≥ 500,000 residents	1.38 (0.79–2.41)	0.3	1.04 (0.58–1.86)	0.9
Marital status				
single	Reference		Reference	
married	0.99 (0.65–1.49)	0.9	0.90 (0.59–1.37)	0.6
informal relationship	2.04 (1.11–3.76)	**0.02**	2.01 (1.08–3.75)	**0.03**
other (divorced or widowed)	0.51 (1.18–1.49)	0.2	0.45 (0.15–1.34)	0.2
Having children <18 y.o.				
Yes	1.18 (0.81–1.70)	0.4		
No	Reference			
Number of household members				
1 (living alone)	Reference			
2	0.78 (0.43–1.41)	0.4		
3 or more	0.87 (0.51–1.51)	0.6		
Characteristics of the workplace				
Private Polish company	Reference			
Public institution	1.49 (0.94–2.35)	0.09		
Private foreign-owned company	1.56 (0.94–2.58)	0.08		
State-owned enterprise	1.10 (0.48–2.53)	0.8		
Non-governmental organization	2.44 (0.31–18.89)	0.4		
Number of employees				
up to 9	Reference		Reference	
10–49	1.58 (0.90–2.78)	0.1	1.42 (0.80–2.53)	0.2
50–249	1.79 (1.03–3.11)	**0.04**	1.54 (0.87–2.72)	0.1
250–500	2.74 (1.25–5.99)	**0.01**	2.61 (1.18–5.80)	**0.02**
More than 500	2.46 (1.37–4.41)	**0.003**	2.07 (1.13–3.80)	**0.02**
Job satisfaction				
Not at all satisfied	1.32 (0.56–3.08)	0.5		
Rather dissatisfied	1.64 (0.87–3.08)	0.1		
Rather satisfied	1.27 (0.79–2.04)	0.3		
Very satisfied	Reference			
Main type of work				
Manual work	0.72 (0.50–1.04)	0.08		
Non-manual work	Reference			
Job position				
Entry-level	Reference			
Specialist	1.21 (0.82–1.79)	0.3		
Manager	1.38 (0.74–2.57)	0.3		
Director-level	1.95 (0.45–8.52)	0.4		
Executive	0.84 (0.28–2.57)	0.8		
Working time arrangement				
Fixed daytime hours	Reference			
Shift work without night shifts	1.00 (0.56–1.79)	0.9		
Shift work including night shifts	0.71 (0.40–1.24)	0.2		
Flexible working hours	1.39 (0.77–2.54)	0.3		
Task-based working time	1.10 (0.53–2.30)	0.8		

Statistically significant values are bolded.

### 3.2 Employees’ expectations regarding different type of employer-provided workplace health promotion activities

Most respondents (35% “definitely yes” and 36.1% “rather yes”) declared that employers should actively support employees’ mental health (Table 3). Among the 16 workplace health promotion analyzed in this study, burnout prevention (35.7%), prevention of spine and musculoskeletal disorders (34.6%), and stress management strategies (34%) were the most frequently indicated by employees (Table 3). In contrast, smoking cessation support (7.7%), diabetes prevention (13%) and obesity prevention (15.7%) were the least frequently indicated WHP activities (Table 3).

**Table 3:** Employees’ expectations regarding different type of employer-provided workplace health promotion activities (*n* = 1,030).

Employees’ expectations regarding different type of employer-provided workplace health promotion (WHP) activities	*n*	%
In your opinion, should employers actively support employees’ mental health (e.g., through consultations with a psychologist or psychiatrist, access to psychotherapy, mobile applications, educational materials, etc.)?		
definitely not	27	2.6
rather not	90	8.7
rather yes	372	36.1
definitely yes	361	35.0
I do not know/ difficult to tell	180	17.5
Which areas of workplace health promotion activities would you like to participate in? Please select all that apply		
*Prevention of chronic diseases and cancer*		
Cancer prevention (including education and self-examination instruction)	173	16.8
Cardiovascular disease prevention	174	16.9
Diabetes prevention	134	13.0
Obesity prevention	162	15.7
*Mental health and workplace well-being*		
Mental health suport	330	32.0
Burnout prevention	368	35.7
Stress management strategies	350	34.0
Work–life balance	315	30.6
*Musculoskeletal health, ergonomics, and pain prevention*		
Prevention of spine and musculoskeletal disorders (e.g., back pain)	356	34.6
Workstation ergonomics	241	23.4
*Lifestyle and behavior change*		
Healthy Nutrition	282	27.4
Healthy lifestyle	281	27.3
Support for physical activity	306	29.7
Support for smoking cessation	79	7.7
*Medical prevention and access to health screening*		
Workplace vaccinations (e.g., influenza vaccination)	170	16.5
Health screening/examinations provided at the workplace and during working hours	253	24.6

### 3.3 Factors determining the belief that employers actively support employees’ mental health

There were significant differences (*p* < 0.05) in the proportion of respondents who declared that employers should actively support employees’ mental health by educational level, type of company, number of employees, and main type of work (manual vs. non-manual) (Table 4).

**Table 4:** Factors determining the belief that employers actively support employees’ mental health (*n* = 1,030).

In your opinion, should employers actively support employees’ mental health—“definitely yes” or “rather yes”						
			Bivariable logistic regression		Multivariable logistic regression	
Variable	%	*p*	OR (95% CI)	*p*	aOR (95% CI)	*p*
Gender						
Female (*n* = 521)	73.5	0.09	1.26 (0.96–1.65)	0.09		
Male (*n* = 509)	68.8		Reference			
Age [years]						
18–24 (*n* = 52)	67.3	0.4	0.99 (0.49–2.00)	0.9		
25–34 (*n* = 317)	74.4		1.40 (0.87–2.24)	0.2		
35–44 (*n* = 284)	72.5		1.27 (0.79–2.04)	0.3		
45–54 (*n* = 266)	68.0		1.02 (0.64–1.64)	0.9		
55–60 (*n* = 111)	67.6		Reference			
Educational level						
Higher education (*n* = 588)	74.8	**0.003**	1.51 (1.15–1.98)	**0.003**	1.32 (0.93–1.86)	0.1
Less than higher (*n* = 442)	66.3		Reference		Reference	
Place of residence						
Rural area (*n* = 351)	70.1	0.8	Reference			
City below 20,000 residents (*n* = 136)	69.9		0.99 (0.64–1.52)	0.9		
City from 20,000 to 99,999 residents (*n* = 198)	71.2		1.06 (0.72–1.55)	0.3		
City from 100,000 to 499,999 residents (*n* = 201)	74.6		1.26 (0.85–1.86)	0.8		
City ≥ 500,000 residents (*n* = 144)	70.1		1.00 (0.66–1.53)	0.9		
Marital status						
Single (*n* = 274)	68.2	0.5	Reference			
Married (*n* = 529)	71.5		1.17 (0.85–1.60)	0.3		
Informal relationship (*n* = 207)	74.4		1.35 (0.90–2.02)	0.1		
Other (divorced or widowed) (*n* = 20)	70.0		1.09 (0.40–2.92)	0.9		
Having children <18 y.o.						
Yes (*n* = 420)	73.8	0.1	1.25 (0.94–1.64)	0.1		
No (*n* = 610)	69.3		Reference			
Number of household members						
1 (living alone) (*n* = 152)	69.7	0.4	Reference			
2 (*n* = 286)	68.5		0.95 (0.62–1.45)	0.8		
3 or more (*n* = 592)	72.8		1.16 (0.79–1.72)	0.5		
Characteristics of the workplace						
Private Polish company (*n* = 513)	66.5	**0.008**	Reference		Reference	
Public institution (*n* = 250)	75.6		1.56 (1.11–2.20)	**0.01**	1.15 (0.80–1.67)	0.5
Private foreign-owned company (*n* = 205)	78.5		1.85 (1.26–2.70)	**0.002**	1.20 (0.78–1.82)	0.4
State-owned enterprise (*n* = 48)	66.7		1.01 (0.54–1.89)	0.9	0.60 (0.31–1.18)	0.1
Non-governmental organization (*n* = 14)	71.4		1.26 (0.39–4.08)	0.7	1.19 (0.36–3.94)	0.8
Number of employees						
Up to 9 (*n* = 119)	52.9	**<0.001**	Reference		Reference	
10–49 (*n* = 240)	69.2		1.99 (1.27–3.14)	**0.003**	1.89 (1.19–2.98)	**0.007**
50–249 (*n* = 289)	69.2		2.00 (1.29–3.10)	**0.002**	1.85 (1.18–2.90)	**0.008**
250–500 (*n* = 108)	74.1		2.54 (1.45–4.45)	**0.001**	2.43 (1.36–4.32)	**0.003**
More than 500 (*n* = 274)	81.8		3.98 (2.48–6.39)	**<0.001**	3.64 (2.16–6.15)	**<0.001**
Job satisfaction						
Not at all satisfied (*n* = 61)	78.7	0.6	1.50 (0.74–3.01)	0.3		
Rather dissatisfied (*n* = 176)	69.3		0.92 (0.57–1.46)	0.7		
Rather satisfied (*n* = 630)	71.0		0.99 (0.68–1.45)	0.9		
Very satisfied (*n* = 163)	71.2		Reference			
Main type of work						
Manual work (*n* = 369)	66.9	**0.03**	Reference	**0.03**	Reference	0.8
Non-manual work (*n* = 661)	73.5		1.37 (1.04–1.81)		1.04 (0.73–1.48)	
Job position						
Entry-level (*n* = 398)	71.1	0.2	Reference			
Specialist (*n* = 462)	71.6		1.03 (0.76–1.38)	0.9		
Manager (*n* = 123)	74.8		1.21 (0.76–1.91)	0.4		
Director-level (*n* = 24)	54.2		0.48 (0.21–1.10)	0.08		
Executive (*n* = 23)	60.9		0.63 (0.27–1.50)	0.3		
Working time arrangement						
Fixed daytime hours (*n* = 603)	69.8	0.8	Reference			
Shift work without night shifts (*n* = 118)	73.7		1.21 (0.78–1.89)	0.4		
Shift work including night shifts (*n* = 99)	73.7		1.21 (0.75–1.96)	0.4		
Flexible working hours (*n* = 138)	73.2		1.18 (0.78–1.79)	0.4		
Task-based working time (*n* = 72)	70.8		1.05 (0.61–1.80)	0.9		

Statistically significant values are bolded.

In the multivariable logistic regression model, number of employees was the only factor significantly associated with employees’ expectations regarding employer-provided mental health support (Table 4). Respondents working in companies with more than 500 employees had over threefold higher odds (aOR: 3.64; 95%CI: 2.16–6.15; *p* < 0.001) of indicating that employers should support employees’ mental health compared with those employed in the smallest companies (≤9 employees) (Table 4).

### 3.4 Employees’ expectations regarding different type of employer-provided workplace health promotion activities by socio-demographic and job-related characteristics

There were significant differences in employees’ expectations regarding different types of employer-provided workplace health promotion activities by socio-demographic and job-related characteristics (Tables 5, 6). Respondents with higher education (*p* < 0.001), those working in companies with more than 500 employees (*p* < 0.001), and non-manual workers (*p* = 0.003) most frequently expressed interest in workplace-based cancer prevention activities compared to other respondent groups (Table 5). Older employes as well as those with higher education (*p* < 0.05) most frequently expressed interest in cardiovascular diseases preventions in workplace compared to other respondent groups. Respondents with children more often reported interest in diabetes prevention (*p* = 0.01). Respondents with higher education (*p* = 0.04), those living in urban areas (*p* < 0.05), respondents working in larger companies (*p* < 0.05), as well as non-manual workers (*p* < 0.05) most frequently declared interest in obesity prevention in workplace compared to other groups of respondents (Table 5).

**Table 5:** Employees’ expectations regarding prevention of chronic diseases and cancer, or mental health and workplace well-being, as a part of workplace health promotion activities (*n* = 1,030).

In your opinion, should employers actively support employees’ mental health—“definitely yes” or “rather yes”																
Variable	Prevention of chronic diseases and cancer								Mental health and workplace well-being							
	Cancer prevention		Cardiovascular disease prevention		Diabetes prevention		Obesity prevention		Mental health suport		Burnout prevention		Stress management strategies		Work–life balance	
	%	*p*	%	*p*	%	*p*	%	*p*	%	*p*	%	*p*	%	*p*	%	*p*
Gender																
Female (*n* = 521)	19.0	0.06	15.2	0.1	11.3	0.1	16.5	0.5	39.7	**<0.001**	41.5	**<0.001**	41.3	**<0.001**	33.6	**0.03**
Male (*n* = 509)	14.5		18.7		14.7		14.9		24.2		29.9		26.5		27.5	
Age [years]																
18–24 (*n* = 52)	13.5	0.7	3.8	**0.04**	13.5	0.7	19.2	0.9	19.2	**0.03**	15.4	**0.01**	25.0	0.5	21.2	0.3
25–34 (*n* = 317)	17.7		14.8		14.2		15.1		35.0		36.6		33.1		32.5	
35–44 (*n* = 284)	18.0		17.3		14.1		16.2		35.2		33.8		35.2		33.1	
45–54 (*n* = 266)	16.9		20.3		11.7		15.8		31.2		38.0		36.5		29.3	
55–60 (*n* = 111)	12.6		19.8		9.9		14.4		23.4		42.3		31.5		26.1	
Educational level																
Higher education (*n* = 588)	20.2	**<0.001**	19.7	**0.005**	14.6	0.08	17.7	**0.04**	37.9	**<0.001**	40.3	**<0.001**	40.3	**<0.001**	35.7	**<0.001**
Less than higher (*n* = 442)	12.2		13.1		10.9		13.1		24.2		29.6		25.6		23.8	
Place of residence																
Rural area (*n* = 351)	14.8	0.4	15.4	0.4	11.4	0.5	9.1	**<0.001**	30.8	0.2	32.2	0.2	30.5	**0.02**	27.1	**0.003**
City below 20,000 residents (*n* = 136)	14.0		13.2		11.0		19.9		26.5		33.8		32.4		22.8	
City from 20,000 to 99,999 residents (*n* = 198)	17.7		20.2		13.6		16.7		35.9		39.9		43.9		37.9	
City from 100,000 to 499,999 residents (*n* = 201)	20.4		17.4		16.4		20.9		30.3		34.8		33.3		28.9	
City ≥ 500,000 residents (*n* = 144)	18.1		18.8		13.2		19.4		37.5		41.7		31.3		38.9	
Marital status																
Single (*n* = 274)	13.1	0.2	17.9	0.6	11.7	0.7	12.8	0.3	33.2	0.6	36.5	0.1	35.8	0.8	30.7	0.1
Married (*n* = 529)	18.7		17.6		14.2		17.6		30.6		34.6		34.0		28.0	
Informal relationship (*n* = 207)	17.4		14.5		12.1		15.5		34.8		39.6		31.4		37.2	
Other (divorced or widowed) (*n* = 20)	10.0		10.0		10.0		10.0		25.0		15.0		35.0		30.0	
Having children <18 y.o.																
Yes (*n* = 420)	18.6	0.2	16.0	0.5	16.2	**0.01**	17.4	0.2	33.1	0.5	33.6	0.2	34.5	0.8	28.3	0.2
No (*n* = 610)	15.6		17.5		10.8		14.6		31.3		37.2		33.6		32.1	
Number of household members																
1 (living alone) (*n* = 152)	14.5	0.7	15.1	0.8	10.5	0.3	11.8	0.3	32.2	0.3	37.5	0.3	32.2	0.6	27.0	0.2
2 (*n* = 286)	17.8		17.5		11.5		15.4		35.7		38.8		36.4		34.3	
3 or more (*n* = 592)	16.9		17.1		14.4		16.9		30.2		33.8		33.3		29.7	
Characteristics of the workplace																
Private Polish company (*n* = 513)	14.0	0.07	16.2	0.8	12.9	0.9	14.2	0.6	27.7	**0.02**	32.7	0.3	31.4	0.06	27.3	0.1
Public institution (*n* = 250)	18.0		17.6		12.4		17.2		38.0		39.2		37.6		33.6	
Private foreign-owned company (*n* = 205)	22.9		18.5		14.1		18.0		35.6		40.0		33.2		36.6	
State-owned enterprise (*n* = 48)	14.6		16.7		12.5		16.7		27.1		33.3		37.5		25.0	
Non-governmental organization (*n* = 14)	14.3		7.1		14.3		7.1		50.0		28.6		64.3		28.6	
Number of employees																
Up to 9 (*n* = 119)	10.1	**<0.001**	12.6	0.07	10.9	0.3	10.1	**0.02**	16.8	**<0.001**	26.1	**0.02**	31.9	**0.04**	18.5	**<0.001**
10–49 (*n* = 240)	12.1		16.3		9.6		11.7		27.9		35.0		34.2		32.1	
50–249 (*n* = 289)	15.9		14.2		14.2		15.9		31.8		32.9		29.4		27.7	
250–500 (*n* = 108)	13.9		16.7		13.9		16.7		33.3		38.9		29.6		24.1	
More than 500 (*n* = 274)	25.9		22.3		15.3		21.2		42.0		42.3		41.2		40.1	
Job satisfaction																
Not at all satisfied (*n* = 61)	23.0	0.5	23.0	0.08	13.1	0.9	14.8	0.3	39.3	**0.03**	44.3	0.3	42.6	0.1	32.8	0.07
Rather dissatisfied (*n* = 176)	16.5		19.9		11.4		11.4		38.6		36.4		36.9		36.4	
Rather satisfied (*n* = 630)	16.8		17.0		13.7		16.2		31.3		35.9		34.0		30.6	
Very satisfied (*n* = 163)	14.7		11.0		12.3		19.0		25.2		31.3		27.6		23.3	
Main type of work																
Manual work (*n* = 369)	12.2	**0.003**	14.6	0.1	12.5	0.7	10.3	**<0.001**	24.1	**<0.001**	29.3	**0.001**	27.4	**<0.001**	26.8	0.05
Non-manual work (*n* = 661)	19.4		18.2		13.3		18.8		36.5		39.3		37.7		32.7	
Job position																
Entry-level (*n* = 398)	15.6	0.4	16.3	0.8	12.8	0.4	15.1	0.7	30.7	**0.02**	36.2	**0.007**	32.7	0.4	31.9	**0.02**
Specialist (*n* = 462)	17.5		17.7		13.4		17.1		34.6		38.7		36.6		33.1	
Manager (*n* = 123)	18.7		17.1		9.8		13.8		35.0		30.9		31.7		22.8	
Director-level (*n* = 24)	20.8		16.7		25.0		16.7		12.5		25.0		29.2		20.8	
Executive (*n* = 23)	8.7		8.7		13.0		8.7		8.7		4.3		21.7		8.7	
Working time arrangement																
Fixed daytime hours (*n* = 603)	17.6	0.4	17.7	0.5	13.6	0.9	16.7	0.9	34.0	0.06	36.8	0.4	36.3	**0.04**	32.2	0.7
Shift work without night shifts (*n* = 118)	15.3		15.3		11.0		13.6		34.7		33.9		37.3		30.5	
Shift work including night shifts (*n* = 99)	10.1		11.1		12.1		15.2		19.2		28.3		21.2		26.3	
Flexible working hours (*n* = 138)	18.8		17.4		13.0		14.5		31.9		39.1		31.2		27.5	
Task-based working time (*n* = 72)	18.1		19.4		12.5		13.9		29.2		33.3		31.9		29.2	

Statistically significant values are bolded.

**Table 6:** Employees’ expectations regarding musculoskeletal health, ergonomics, and pain prevention, lifestyle and behavior change, or mental health, or medical prevention and access to health screening, as a part of workplace health promotion activities (*n* = 1,030).

In your opinion, should employers actively support employees’ mental health—“definitely yes” or “rather yes”																
Variable	Musculoskeletal health, ergonomics, and pain prevention				Lifestyle and behavior change								Medical prevention and access to health screening			
	Prevention of spine and musculoskeletal disorders		Workstation ergonomics		Healthy nutrition		Healthy lifestyle		Support for physical activity		Support for smoking cessation		Workplace vaccinations		Health screening/ examinations provided at the workplace and during working hours	
	%	*p*	%	*p*	%	*p*	%	*p*	%	*p*	%	*p*	%	*p*	%	*p*
Gender																
Female (*n* = 521)	41.3	<0.001	26.3	0.03	30.1	0.04	30.7	0.01	32.4	0.05	6.5	0.2	15.5	0.4	26.9	0.08
Male (*n* = 509)	27.7		20.4		24.6		23.8		26.9		8.8		17.5		22.2	
Age [years]																
18–24 (*n* = 52)	11.5	0.001	17.3	0.04	25.0	0.6	25.0	0.2	30.8	0.6	11.5	0.1	9.6	0.07	25.0	0.3
25–34 (*n* = 317)	31.2		28.1		28.7		31.2		31.9		7.6		15.5		24.6	
35–44 (*n* = 284)	39.8		25.4		28.9		26.4		30.3		4.6		13.7		26.1	
45–54 (*n* = 266)	37.2		18.4		27.1		27.4		28.9		8.6		19.2		20.3	
55–60 (*n* = 111)	35.1		19.8		21.6		18.9		23.4		11.7		23.4		30.6	
Educational level																
Higher education (*n* = 588)	41.3	<0.001	29.8	<0.001	32.2	0.002	31.1	0.001	34.0	<0.001	6.6	0.1	18.2	0.09	27.7	0.007
Less than higher (*n* = 442)	25.6		14.9		33.4		22.2		24.0		9.0		14.3		20.4	
Place of residence																
Rural area (*n* = 351)	31.6	0.4	19.4	0.1	23.4	0.1	25.1	0.6	23.9	0.06	7.7	0.7	13.7	0.2	22.5	0.7
City below 20,000 residents (*n* = 136)	32.4		22.8		25.0		27.9		30.9		5.9		14.7		24.3	
City from 20,000 to 99,999 residents (*n* = 198)	37.9		25.8		31.3		31.3		32.3		8.1		17.7		23.7	
City from 100,000 to 499,999 residents (*n* = 201)	34.3		23.9		32.8		27.9		33.8		9.5		17.9		26.4	
City ≥ 500,000 residents (*n* = 144)	39.6		29.9		26.4		25.7		33.3		6.3		21.5		28.5	
Marital status																
Single (*n* = 274)	29.9	0.3	23.4	0.8	26.3	0.3	25.2	0.4	29.2	0.4	7.7	0.9	20.1	0.2	21.5	0.1
Married (*n* = 529)	35.9		22.5		26.3		27.8		28.2		7.2		14.7		25.0	
Informal relationship (*n* = 207)	37.7		26.1		32.4		30.0		33.3		8.7		15.5		29.0	
Other (divorced or widowed) (*n* = 20)	30.0		20.0		20.0		15.0		40.0		10.0		25.0		10.0	
Having children <18 y.o.																
Yes (*n* = 420)	37.4	0.1	22.4	0.5	25.7	0.3	30.0	0.1	30.5	0.7	6.0	0.09	12.6	0.005	25.5	0.6
No (*n* = 610)	32.6		24.1		28.5		25.4		29.2		8.9		19.2		23.9	
Number of household members																
1 (living alone) (*n* = 152)	32.2	0.7	23.7	0.04	27.0	0.03	19.1	0.04	29.6	0.9	5.9	0.2	21.1	0.08	21.1	0.5
2 (*n* = 286)	36.4		28.7		33.2		30.4		29.7		10.1		18.5		24.1	
3 or more (*n* = 592)	34.3		20.8		24.7		27.9		29.7		6.9		14.4		25.7	
Characteristics of the workplace																
Private Polish company (*n* = 513)	29.2	0.01	21.1	0.2	27.5	0.01	26.3	0.5	27.9	0.2	7.2	0.09	14.6	0.5	23.0	0.6
Public institution (*n* = 250)	39.2		23.6		25.2		27.2		33.6		5.2		17.6		27.6	
Private foreign-owned company (*n* = 205)	40.5		28.8		29.3		31.7		28.3		11.7		20.0		24.4	
State-owned enterprise (*n* = 48)	37.5		20.8		27.1		20.8		29.2		6.3		16.7		22.9	
Non-governmental organization (*n* = 14)	50.0		35.7		35.7		21.4		50.0		14.3		14.3		35.7	
Number of employees																
Up to 9 (*n* = 119)	28.6	0.004	16.0	0.03	18.5	0.01	21.8	0.4	21.8	0.04	4.2	0.6	10.9	0.006	16.8	0.01
10–49 (*n* = 240)	28.7		20.8		22.1		25.4		27.9		7.1		13.3		22.9	
50–249 (*n* = 289)	32.5		21.8		31.5		27.3		27.3		8.7		15.9		22.8	
250–500 (*n* = 108)	38.0		30.6		26.9		32.4		36.1		7.4		13.9		22.2	
More than 500 (*n* = 274)	43.1		27.7		31.8		29.2		34.7		8.8		23.4		32.1	
Job satisfaction																
Not at all satisfied (*n* = 61)	27.9	0.2	32.8	0.3	21.3	0.6	26.2	0.9	29.5	0.7	6.6	0.5	29.5	0.02	23.0	0.7
Rather dissatisfied (*n* = 176)	36.9		22.7		27.8		26.1		32.4		9.1		17.6		27.3	
Rather satisfied (*n* = 630)	36.0		23.2		28.4		27.9		28.4		8.1		14.6		24.6	
Very satisfied (*n* = 163)	28.8		21.5		25.2		26.4		31.9		4.9		17.8		22.1	
Main type of work																
Manual work (*n* = 369)	29.3	0.008	14.6	<0.001	23.3	0.03	22.2	0.006	23.8	0.002	9.8	0.06	15.4	0.5	22.0	0.1
Non-manual work (*n* = 661)	37.5		28.3		29.7		30.1		33.0		6.5		17.1		26.0	
Job position																
Entry-level (*n* = 398)	37.7	0.003	21.1	0.3	25.1	0.1	26.6	0.09	30.2	0.04	10.1	<0.001	16.3	0.9	23.9	0.2
Specialist (*n* = 462)	35.9		26.2		31.0		30.3		32.5		5.2		16.9		27.5	
Manager (*n* = 123)	27.6		22.8		23.6		22.8		22.0		4.1		15.4		18.7	
Director-level (*n* = 24)	20.8		20.8		29.2		20.8		29.2		20.8		20.8		20.8	
Executive (*n* = 23)	4.3		13.0		13.0		8.7		8.7		21.7		13.0		13.0	
Working time arrangement																
Fixed daytime hours (*n* = 603)	38.3	0.04	26.0	0.2	29.4	0.2	28.2	0.3	32.5	0.04	6.6	0.4	16.4	0.4	26.0	0.5
Shift work without night shifts (*n* = 118)	30.5		20.3		20.3		22.9		24.6		7.6		22.0		25.4	
Shift work including night shifts (*n* = 99)	26.3		16.2		21.2		20.2		18.2		9.1		13.1		24.2	
Flexible working hours (*n* = 138)	28.3		21.7		29.7		31.2		31.2		8.7		14.5		20.3	
Task-based working time (*n* = 72)	33.3		19.4		26.4		29.2		27.8		12.5		16.7		19.4	

Female respondents, those with higher education, employees working in companies with more than 500 employees, non-manual workers, and individuals in entry-level or specialist positions more frequently declared interest in WHP activities focused on mental health and workplace well-being (*p* < 0.05), compared to other employee groups (Table 5).

Female respondents (*p* < 0.001), employees aged ≥25 years (*p* < 0.05), those with higher education (*p* < 0.001), individuals working in private foreign-owned companies or NGOs (*p* = 0.01), employees from companies with more than 500 employees (*p* = 0.004), non-manual workers (*p* = 0.008), respondents in entry-level or specialist role (*p* = 0.003), and those working fixed daytime hours (*p* = 0.04) most frequently reported interest in workplace activities aimed at prevention of spine and musculoskeletal disorders compared with other respondent groups (Table 6).

Interest in workstation ergonomics was more common among women (*p* = 0.03), employees aged 25–44 years (*p* < 0.05), respondents with higher education (*p* < 0.001), those living with one other household member (*p* = 0.04), those working in companies with at least 250 employees (*p* = 0.03), and non-manual workers (*p* < 0.001) (Table 6).

Interest in lifestyle-oriented WHP activities (healthy nutrition or healthy lifestyle) was higher among women, respondents with higher education, those living with one other household member, and non-manual workers (*p* < 0.05) (Table 6). In addition, employees working in non-governmental organizations and those employed in companies with more than 500 employees more frequently (*p* < 0.05) indicated healthy nutrition as preferred WHP activity.

Respondents with higher education (*p* = 0.001), those working in companies with at least 250 employees (*p* = 0.04), non-manual workers (*p* = 0.002), entry-level or specialist roles (*p* = 0.04), and respondents working fixed daytime hours (*p* = 0.04) more often reported interest in WHP activities aimed at supporting physical activity (Table 6). Directors and executives more often indicated that employers should provide smoking cessation support for employees who smoke compared with respondents in lower job positions (*p* < 0.001).

Support for workplace vaccinations was more frequently reported by respondents without children (*p* = 0.005), those employed in companies with more than 500 employees (*p* = 0.006), and individuals who were not satisfied with their job (Table 6). Moreover, respondents with higher education (*p* = 0.007) and those working in companies with more than 500 employees (*p* = 0.01) more frequently declared interest in health screening/examinations provided at the workplace and during working hours (Table 6).

## 4 Discussion

This study provides a comprehensive characteristics of employees’ expectations regarding employer-provided workplace health promotion and mental health support in Poland. The findings indicate that employees strongly support the implementation of workplace health promotion (WHP) activities and mental health support programs. Employees most frequently prioritized burnout prevention, musculoskeletal disorder prevention, and stress-management strategies, indicating that well-being and functional capacity are major expectations towards WHP. There were significant sociodemographic and job-related differences in employees’ interest in particular scopes of WHP activities, which underscores the need to assess employees’ needs and preferences when planning WHP activities.

Employer-provided WHP interventions may improve health outcomes, support lifestyle changes, reduce absenteeism, and presenteeism in the workplace (11, 12). WHP interventions differ across the companies, with the strong role of managers and company leaders as initiators and drivers of WHP implementation in the company (31, 32). There is a limited number of scientific papers on WHP in Poland (19–24). Most of them are affiliated with the Nofer Institute of Occupational Medicine – the leading scientific institution tasked with occupational medicine in Poland, which in 2015 and 2017 conducted studies on companies employing ≥50 people (19–23). Puchalski and Korzeniowska showed that in 2015, half of companies undertook voluntary actions for workers health, with medical care, physical activity support work environment adjustment as the most common WHP interventions (20). As reported by the employees, company image, productivity improvement, and cost reduction were the most common motivations for WHP implementation (20). Suchańska and Marcinkiewicz reported that offering private medical care for employees is considered by employers as the most common health-related intervention in the workplace (23).

The results of this study suggest that employers may not fully meet employees’ expectations regarding WHP. High support for WHP promotion declared by full-time employees in Poland also suggests that employees may consider health-related activities as a standard non-wage benefit offered by the employer in the current labor market (33). The differences between the percentage of companies that declare the implementation of WHP interventions (20, 23) and the percentage of employees expecting employer-provided WHP suggest that a significant percentage of employers do not meet employees’ expectations regarding WHP.

It is estimated that over one-fifth of employers in Europe report symptoms of burnout, and around 40% of workers report severe time pressure and work-related stress (34). Due to the growing burden of risk factors for mental well-being, interventions aimed at strengthening employee mental health are one of the leading trends in WHP interventions worldwide (1, 10–12). Companies are developing mental health support strategies using stress-management programs, psychological counseling, peer support, and digital tools to support employees’ mental health and well-being (11–13). High interest in mental health support observed in this study may reflect increasing psychological demands and work-related stress experiences by employees.

An analysis of employer-based health plans in Poland showed significant insurance limitations and policy gaps related to mental health support and treatment (24). The offer for mental health treatment through employee medical plans is strongly limited to the number of visits and scope of services (24). Therefore there is a need to strengthen mental health support offered by employers in Poland.

Out of 16 different scopes of WHP interventions analyzed in this study, employees most frequently prioritized burnout prevention, musculoskeletal disorder prevention, and stress-management strategies. Findings from this study suggest that well-being and functional capacity are major expectations of WHP. Musculoskeletal disorders are one of the leading causes of sick leaves, so WHP interventions focused on this topic may significantly reduce absenteeism (35, 36). However, there were significant differences in employees’ expectations regarding different types of employer-provided workplace health promotion activities by socio-demographic and job-related characteristics. This observation emphasizes the importance of assessing employee needs and expectations before implementing WHP interventions (37). Matching WHP interventions to employee expectations is crucial to the effectiveness of WHP interventions and ensuring a high participation rate (5–8, 37).

In multivariable logistic regression, educational level, place of residence, and marital status were sociodemographic characteristics associated with employees’ expectations towards employer-provided workplace health promotion. Full-time employees with higher education were more likely to report that employers should provide WHP interventions. Individuals with higher education are often considered to have higher health literacy levels (38). In Organization for Economic Co-operation and Development (OECD) countries, individuals with higher education have better health and life expectancies compared to those without higher education (39). This study suggests that individuals with higher education may have higher expectations regarding the workplace health promotion and health-related non-wage benefits that may reflect greater awareness of the preventive health interventions.

Individuals living in cities with populations of 20,000 to 99,999 residents, compared to those living in rural areas, were also more likely to report that employers should provide workplace health promotion interventions. This may result from the fact that those living in medium-sized cities may have limited access to healthcare services, so they expect health-related activities from their employers. Moreover, large companies are often located in medium-sized cities, e.g., on communication routes or in the vicinity of large cities, which may also translate into employee expectations—a larger employer may be perceived as having greater opportunities to provide employee benefits (3, 21, 22, 31).

In this study, individuals in informal relationships were more likely to declare that employers should provide WHP activities. This observation may result from broader social changes (declining marriage rates, changes in family models) and should be analyzed carefully.

Number of employees (company size) was the only job-related factor significantly associated with employees’ expectations towards both employer-provided workplace health promotion and mental health support (5–7, 37). Individuals working in companies with at least 250 employees were more likely to report that employers should provide WHP interventions. Odds of reporting expectations towards employer-provided mental health support increased with the number of employees. These observations suggest that employees may perceive larger companies as having more resources to implement WHP compared to small businesses. Larger companies may have better organizational resources—structured HR departments, budgets, procedures, established network of vendors, and the ability to scale programs (5–7, 37). In Poland, previous studies on WHP were conducted in companies employing > 50 employees (19–23). On the other hand, we can assume that in larger organizations, employees may feel the impact of their work environment on their physical and mental health more acutely. This may be particularly acute when faced with the pressure, stress, and multitasking of large organizations, which impacts mental health and well-being.

Regarding mental health support, respondents working in companies with 10 or more employees were more likely to declare interest in mental health support, compared to those working in companies with fewer than nine employees. This demonstrates that even in smaller companies, mental health is important and should be considered as part of a WHP strategy.

Workplace health promotion activities focused on the prevention of chronic diseases and cancer (a list of diseases such as: diabetes, obesity, cancer and cardiovascular diseases) aroused less interest among employees than activities aimed at lifestyle and behavior change (such as support for physical activity, healthy nutrition and healthy lifestyle). This may suggest that in planning WHP, the method of communication, naming and perception of a given topic as something negative (presence of disease) or positive (health-promoting change) are also important.

Although the study sample covered employees from across Poland, the results should be interpreted in the context of the national labor market and organizational culture. Therefore, generalization to other countries should be made cautiously, particularly in healthcare systems and labor market environments that differ from those in Poland.

In addition to mental health support, workplace health promotion programs may also play an important role in the prevention of chronic diseases such as cardiovascular diseases, diabetes, and obesity. These conditions represent a major public health burden worldwide and are strongly influenced by lifestyle factors including physical activity, nutrition, and stress management (25). Workplace interventions promoting healthy behaviors may therefore contribute not only to improved employee well-being but also to the prevention of chronic diseases and reduction of long-term healthcare costs.

### 4.1 Practical implications

The findings from this study have several practical implications for both employers and public health professionals. This study revealed that employees expect employers to provide workplace health promotion and mental health support. These findings clearly show which scope of WHP interventions should be considered as the priority for implementation in the workplace. However, this study also revealed socio-demographic and job-related differences in public expectations towards particular types of WHP interventions, so employers should analyze employees’ expectations and needs, e.g., using questionnaires and focus groups. High employee interest in WHP and mental health support in the workplace suggests that policymakers should implement organizational and fiscal solutions that facilitate the implementation of WHP and encourage employers (e.g., through tax relief) to implement such activities.

### 4.2 Limitations

This cross-sectional study was conducted using the CAWI technique, so individuals without internet access (approximately 4% of households in Poland (27)) were excluded from this study. The study sample included adults aged 18–60 years with full-time employment under an employment contract, so results cannot be generalized to the entire population of working adults. Nevertheless, full-time employment under an employment contract is the most common form, and employment and age over 60 is retirement for women. The data in this study were self-declared, so recall bias may occur. Respondents were asked about attitudes towards the 16 most common WHP topics that were selected based on a literature review. Further studies may include other types of WHP interventions. Employees’ expectations of WHP interventions may not fully reflect the potential utilization of WHP interventions.

## 5 Conclusion

Most full-time employees in Poland expect employers to implement workplace health promotion and to provide mental health support. Expectations varied by sociodemographic and job-related characteristics, with company size emerging as the key factor determining expectations towards WHP. These findings indicate high acceptability of WHP among employees and point to the need for scalable approaches that also reach workers in smaller companies. Sociodemographic and job-related differences observed in this study indicate that employers should assess employees’ needs and preferences before designing and implementing WHP activities.

## Data Availability

The raw data supporting the conclusions of this article will be made available by the authors, without undue reservation.
