# Supplementary material for: Employee expectations towards employer-provided workplace health promotion and mental health support: a 2025 cross-sectional survey
Source: Front Public Health. 2026 Apr 9;14:1806781. doi: 10.3389/fpubh.2026.1806781 (PMC13102647; doi:10.3389/fpubh.2026.1806781)
Supplement: Supplementary file 1 [file Supplementary_file_1.DOCX]

**Supplementary File S1. Study Questionnaire**

What is your gender?

[rotation]

- Female
- Male

What is your age? Select your age range:

- 18-24 years
- 25-34 years
- 35-44 years
- 45-60 years

Where do you live? Select the size of the town or city you live in.

- Rural area
- Small town (up to 20,000 inhabitants
- Medium-sized town (21,000 to 99,000 inhabitants)
- Large city (100,000 to 500,000 inhabitants)
- Varietal city (over 500,000 inhabitants)

What is your current main employment status?

[Rotation – multiple response]

- Employed full-time under a contract of employment
- Employed part-time under a contract of employment => terminate
- Working under a civil-law contract (contract of mandate / contract for specific task) => terminate
- Self-employed (own business) => terminate
- Retired / disability pension recipient => terminate
- Pupil/student => terminate
- Homemaker => terminate
- Other (please specify) [not rotated] => terminate

[ONLY respondents who are employed full-time under a contract of employment and are aged ≤60 years continue; all others terminate.]

[All employed respondents]

Which description best fits the company or institution where you currently work?

[Rotation – single response]

- Private Polish company
- Public institution (e.g., public office, school, military, police, public healthcare provider, etc.)
- Private foreign-owned company
- State-owned enterprise
- Non-governmental organisation (NGO; association/foundation)

Approximately how many employees does the company or institution where you currently work have?

- Up to 9 employees
- 10–49 employees
- 50–249 employees
- 250–500 employees
- More than 500 employees

Are you satisfied with your current job?

- Not at all satisfied
- Rather dissatisfied
- Rather satisfied
- Very satisfied

[Topic change]

Workplace health promotion (WHP) is a coordinated set of activities and strategies at the workplace (offered by the employer) to encourage the health and safety of all employees. WHP is not occupational medicine, but additional activities such as promoting a healthy lifestyle, especially a healthy diet, physical activity, and disease prevention.

Does your employer undertake workplace health promotion (WHP) activities aimed at improving employees’ health?

[Rotation]

- - Yes, regularly
  - Yes, occasionally / one-off
  - No
  - Don’t know / hard to say [not rotated]

[If the employer undertakes WHP activities]

Which workplace health promotion activities are provided by your employer?

Please select all that apply or specify other.

[Rotation – multiple response]

- - Access to private medical care
  - Life insurance (e.g., benefits in case of cancer, surgery, hospitalisation, childbirth)
  - Sports card or access to a sports facility
  - Meal card or subsidised meals at work
  - Free fruit and healthy snacks available at the workplace
  - Access to a psychologist/psychiatrist (in addition to the medical package; e.g., via a dedicated hotline, app, or online portal)
  - On-site vaccinations (e.g., influenza vaccination)
  - On-site health checks (e.g., blood sampling, blood pressure measurement)
  - Educational initiatives (webinars, lectures, training sessions, workshops)
  - Group participation in sports events (e.g., races) or team sports with colleagues
  - Promotion of active commuting by bicycle (e.g., secure bicycle parking and changing facilities)
  - On-site massages or physiotherapy services
  - Other (please specify) [not rotated]

[If the employer undertakes WHP activities]

To what extent do you agree with the following statements about workplace health promotion activities organised by your employer?

[Horizontal slider scale: Strongly disagree – Rather disagree – Rather agree – Strongly agree – Don’t know / hard to say]

[Item rotation]

- - Before implementing WHP activities, the employer assesses employees’ needs (e.g., via a survey) and adapts actions to the results.
  - After implementing WHP activities, the employer assesses employees’ satisfaction (e.g., via a survey).
  - The WHP activities implemented by my employer meet my needs.
  - The WHP activities implemented by my employer improve my health and well-being.
  - The WHP activities implemented by my employer increase my engagement at work.

[If the employer undertakes WHP activities]

Do you use the workplace health promotion activities offered by your employer?

- Never
- Rarely
- Often
- Very often

[If the employer undertakes WHP activities]

[P16]

What makes it difficult for you to participate in workplace health promotion activities organized by your employer?

Please select all relevant factors.

[Rotation – multiple response]

- Lack of time
- Lack of information or information provided too late
- Limited availability of places (participant cap); difficult to register
- Topics are not interesting / do not match my needs
- There are too many such activities and I feel fatigued by them
- Concerns about privacy and confidentiality of my personal information
- Unequal access (e.g., exclusion due to shift schedule, location, lack of a corporate email)
- Better offer for white-collar employees than for manual workers
- I do not believe such activities are effective
- Lack of motivation on my part / I am not interested
- Other (please specify) [not rotated]
- I do not see any barriers [not rotated]

[All respondents]

In your opinion, should the employer implement workplace health promotion (WHP) activities?

- yes
- no

[All respondents]

In your opinion, should employers actively support employees’ mental health (e.g., through consultations with a psychologist or psychiatrist, access to psychotherapy, mobile apps, educational materials, etc.)?

- - Strongly no
  - Rather no
  - Rather yes
  - Strongly yes
  - Don’t know / hard to say

[All respondents]

Which areas of workplace health promotion would you like to participate in?

Please select all that apply or specify other.

[Rotation – multiple response]

- - - Cancer prevention (education, self-examination instruction)
    - Prevention of spine and musculoskeletal disorders (e.g., back pain)
    - Healthy nutrition
    - Healthy lifestyle
    - Mental health support
    - Burnout prevention
    - Obesity prevention
    - Diabetes prevention
    - Cardiovascular disease prevention
    - Stress management methods
    - Support for smoking cessation
    - Workstation ergonomics
    - Vaccinations available at the workplace (e.g., influenza vaccination)
    - Support for physical activity
    - Organisation of medical examinations at the workplace and during working hours
    - Work–life balance
    - Other (please specify) [not rotated]
    - I am not interested in workplace health promotion activities at all [not rotated]

[Topic change]

What type of work do you mainly do?

[Rotation]

- Mainly manual work
- Mainly desk/knowledge work

Which description best fits your position?

[Rotation]

- - - Entry-level (I work under the supervision of others)
    - Specialist (I have supervisors but work largely independently)
    - Managerial (I supervise the work of at least two people)
    - Director-level (I manage a department)
    - Executive (I manage a company or institution)

What is your working time arrangement?

- - Fixed daytime hours
  - Shift work without night shifts
  - Shift work including night shifts
  - Flexible working hours
  - Task-based working time

In which sector/industry do you work? Please select the most appropriate description.

[Rotation – multiple response]

- - Agriculture, forestry, hunting, and fishing
  - Mining and quarrying
  - Manufacturing
  - Electricity, gas, steam, and air conditioning supply
  - Water supply; sewerage, waste management and remediation activities
  - Construction
  - Wholesale and retail trade; repair of motor vehicles and motorcycles
  - Transportation and storage
  - Accommodation and food service activities
  - Information and communication
  - Financial and insurance activities
  - Real estate activities
  - Professional, scientific and technical activities
  - Administrative and support service activities
  - Public administration and defence; compulsory social security
  - Education
  - Human health and social work activities
  - Arts, entertainment and recreation
  - Other service activities
  - Activities of households as employers; undifferentiated goods- and services-producing activities of households for own use
  - Extraterritorial organisations and bodies (e.g., embassies, organisations with immunities, armed forces of other countries, etc.)
  - Other (please specify) [not rotated]

What is your current level of education (highest completed level)?

- Primary or lower secondary
- Basic vocational education
- Secondary education
- Post-secondary (non-tertiary) education
- Bachelor’s degree
- Higher education completed (Master’s degree or equivalent)

What is your marital status? Select:

- single
- married
- in a civil partnership
- other (please specify)

Do you have children under 18?

- yes
- no

How many people live in your household?

- I live alone
- 2
- 3
- 4
- 5 or more
